# Die Rolle der Schulleitung in der berufspraktischen Ausbildung von Lehrerinnen und Lehrern

**DOI:** 10.1007/s35834-025-00517-1

**Published:** 2025-11-18

**Authors:** Mirjam Kocher, Anna Locher, Julia Košinàr

**Affiliations:** Forschung und Entwicklung, Pädagogische Hochschule Zürich, Lagerstrasse 2, 8090 Zürich, Schweiz

**Keywords:** Schulleitung, Ausbildung von Lehrpersonen, Berufspraktische Ausbildung, Praxislehrpersonen, Mentorin und Mentor, School principals, Teacher education, Vocational training, Mentor teacher

## Abstract

Der vorliegende Beitrag befasst sich mit der Rolle von Schulleitungen der Volksschule in der Ausbildung von zukünftigen Lehrpersonen und fragt nach 1.) deren Kriterien für die Auswahl und Empfehlung von Praxislehrpersonen und den eingeschätzten Qualifizierungsnotwendigkeiten für die Praxislehrpersonentätigkeit, sowie 2.) den Aufgaben und Anforderungen, die die Schulleitungen der Praxislehrpersonenrolle und -tätigkeit und sich selbst im Ausbildungskontext zuschreiben. Hierfür wurden 19 Schulleiter:innen der Deutschschweiz mittels Interviews befragt. Nach der inhaltsanalytischen Auswertung des Datenmaterials zeigen die Ergebnisse zusammenfassend, dass das konkrete Ausbildungshandeln der Praxislehrpersonen den Schulleitungen in der Regel wenig zugänglich ist. Es werden differenzierte Auswahlkriterien für Praxislehrpersonen von den Schulleitungen genannt, wobei sich diese im Wesentlichen auf die Unterrichtsqualität der Lehrpersonen beziehen. Weder geraten die Vielfalt der Aufgaben einer Praxislehrperson als Ausbildner:in noch derer Weiterentwicklung und Unterstützung in ihren Fokus. Weiterhin sehen sie sich selbst kaum in einer aktiven Rolle in der berufspraktischen Ausbildung zukünftiger Lehrpersonen.

## Abstract (en)

The present article deals with the role of school principals in the training of future teachers and addresses 1) the criteria for selecting mentor teachers and the qualification requirements for the mentor teacher role, as well as 2) the tasks and demands that they attribute to the mentor teacher role and activity and to themselves in the training context. For this purpose, 19 school principals from German-speaking Switzerland were interviewed. After content analysis of the data, the results show that the specific training actions of mentor teachers are generally not very accessible to the school principals. Differentiated selection criteria for mentor teachers are mentioned by the school principals, with criteria related to teaching quality being reported most frequently. Neither the diversity of tasks of a mentor teacher nor their further development and support come into their focus. They also see themselves hardly in an active role in the practical training of future teachers.

## Einleitung

In der einphasigen Schweizer Lehrer:innenausbildung haben Praktika vor Ort in Schulen einen zentralen Stellenwert. Sie gewährleisten mit der unterrichtspraktischen Bewährung der Studierenden einen erfolgreichen Berufseinstieg nach dem 3 bzw. 4,5-jährigen Studium^1^. Die Studierenden werden während der Praktika, die, je nach Konzeption der Pädagiogischen Hochschule (PH), zwischen 3 Wochen und 10 Monaten dauern, von Praxislehrpersonen (PLP^99^) begleitet. Den PLP kommt dabei als Modell, Beratende und Beurteilende eine zentrale Rolle in der Ausbildung zu (z. B. Festner et al. 2020; Košinár et al. 2019; Baer et al. 2021).

Die PLP werden für ihre Tätigkeiten üblicherweise von der PH vertraglich entlöhnt. Damit sind PLP zwei Institutionen (der PH und der Schule vor Ort) verpflichtet und nehmen somit eine Doppelrolle als Ausbildner:in und als Lehrperson ein (Hascher und Moser 2001, Košinár 2023). Die Hochschule stellt Anforderungen an die PLP bezüglich deren Umsetzung und Begleitung in den verschiedenen Praktika, wobei sich diese je nach PH unterscheidet (Košinár 2023). Die PHs streben teilweise mit der Einführung von Partner- oder Kooperationsschulen (Kreis et al. 2023) eine Intensivierung der Zusammenarbeit an, indem regelmässige Treffen zwischen Dozierenden der PH und PLP organisiert werden (Fraefel et al. 2017; Reusser und Fraefel 2017). Obwohl die PLP von den PHs entlöhnt werden, liegt die personelle Führung der PLP nicht bei der Hochschule, sondern bei den Schulleitenden (SL). Mit dieser Verantwortung wird die SL eine weitere wichtige Akteursgruppe in der berufspraktischen Ausbildung. Ihre Rolle und Funktion darin ist bislang jedoch kaum Gegenstand von Forschung (Scherrer 2020, S. 177). Insgesamt kann aus Studien zur Schulentwicklung und Qualitätssicherung, welche den Einfluss der SL auf Schule und Kollegium empirisch belegt (bspw. Betschart et al. 2022), geschlussfolgert werden, dass SL auch eine Bedeutung in der qualitativ guten Ausgestaltung der schulpraktischen Ausbildung an ihrer Schule zukommt.

Der vorliegende Beitrag nimmt sich dem Forschungsdesiderat an und erfasst das subjektive Rollenverständnis von SL in der Ausbildung von Studierenden an ihrer Schule^2^. Zuerst wird auf die Personalentwicklung, auf die berufspraktische Ausbildung von Lehrpersonen und auf die Aufgabenbereiche von SL in der Ausbildung von Lehrpersonen eingegangen. Nach der Erläuterung der Fragestellungen, Design und Methodik der Studie werden die Ergebnisse zum Personalentwicklungsverständnis von SL, zu Anforderungen an PLP aus der Sicht der SL sowie zu Funktionen und Rolle von SL in der Ausbildung von Lehrpersonen dargelegt. Die Diskussion geht mit Blick auf Desiderate auf die Ergebnisse ein.

## Theoretischer Hintergrund und Forschungsstand

Mit der Forderung nach mehr Schulautonomie in den 1990er-Jahren im deutschsprachigen Raum wurden in der Schweiz im Sinne eines New Public Managements erstmals Schulleitungen eingeführt (bspw. Dubs 1996). Daher sind sie in der Schweiz eine junge Profession und seit ihrer Etablierung hat sich das Berufsverständnis bereits stark entwickelt (Anderegg und Breitschaft 2020). Heute werden nicht mehr Themen der Autonomiegestaltung von Schulen in den Vordergrund gestellt, viel mehr sind betriebswirtschaftliche Führungsstrukturen ins Zentrum gerückt worden und damit Prinzipien der Führung und Leitung des Schulpersonals (Hangartner und Svaton 2016), das auch die PLP als Lehrpersonen des Schulkollegiums einschliesst. Zahlreiche Studien gehen auf das Führungs- und Steuerungshandeln bzw. auf das Schulmanagement und auf den Einfluss von SL einschliesslich ihres Führungsverhaltens auf Schule und Kollegium ein (bspw. Überblick: Brauckmann und Eder 2019; Bonsen 2016; Windlinger et al. 2020; Freisler-Mühlemann et al. 2021). Auch wurde ihr Einfluss z. B. auf die Gesundheit des Lehrpersonals (z. B. Betschart et al. 2022; Schoch et al. 2021) oder auf das Lernen der Schüler:innen (Tulowitzki et al. 2023) nachgewiesen. Allen angeführten Studien gemeinsam ist, dass Aspekte der Ausbildung angehender Lehrpersonen nicht thematisiert werden.

### Personalentwicklung als entscheidender Faktor für Schulentwicklung und Spezialisierungen wie die PLP-Tätigkeit

Mit der zunehmenden Autonomie der einzelnen Schulen und der zentralen Rolle von Lehrpersonen für das Lernen der Schüler:innen stellt die Personalentwicklung einen wichtigen Aspekt von Schulqualität dar (Böckelmann und Mäder 2018; Terhart 2016). Personalentwicklung umfasst insgesamt die Auswahl von geeigneten Personen, die Vorbereitung auf die Aufgabe, die Weiterbildung der Fähigkeiten der Mitarbeitenden und die Erhaltung ihrer Motivation (Terhart 2016, S. 279). Diese ist im Schulkontext besonders notwendig, und es ist

„im Interesse zweckgerichteter und wirksamer Arbeitsabläufe der Personalauswahl, dem Personaleinsatz und der Personalentwicklung maximale Aufmerksamkeit zu schenken. Denn die Arbeit in Bildungseinrichtungen ist sehr stark personengebunden, also in Ablauf wie Ergebnis entscheidend von den Personen abhängig, die dort arbeiten. Schulen gehören zu den *people-processing* bzw. *people-changing-institutions*; in solchen Institutionen spielt der Faktor Personal eine ganz entscheidende Rolle, weil das Personal nicht einfach nur Technologien einsetzt …“ (Terhart 2016, S. 279).

Die Personalentwicklung umfasst dabei nicht einzelne Qualifizierungsmassnahmen, sondern eine zielgerichtete, längerfristige Planung von Einzel- und Teamweiterbildungen(-entwicklungen) im Hinblick auf die Entwicklung der Schule als Organisation (Böckelmann und Mäder 2018). Hierfür sind neben der Berücksichtigung von individuellen Weiterbildungswünschen auch auf die Organisation ausgerichtete Spezialisierungen notwendig, um den vielfältigen Anforderungen, die heute an Schulen gestellt werden, zu genügen. Im Hinblick auf die berufspraktische Ausbildung von angehenden Lehrpersonen kann die Rolle der PLP als Spezialisierung angesehen werden (LCH Leitbild, 2024)^3^. Dies wird zusätzlich verstärkt vor dem Hintergrund der Implementierung intensivierter Kooperationsformen zwischen Schweizer PH’s und Schulen (z. B. in Form von Kooperationsschulen, Praxiszentren, Partnerschulen) (vgl. Kreis et al. 2023; Košinár et al. 2019). Mit der Einführung von Partnerschulen bspw. im Bildungsraum Nordwestschweiz 2017 oder den Kooperationsschulen bzw. Praxiszentren an der PH Zürich haben SL explizit eine Verantwortung für die Ausbildung angehender Lehrpersonen übernommen, indem sie ausreichend Praktikumsplätze für Studierende und ein Team von PLP zusammenstellen. Auch nehmen SL eine Aufgabe der Personalentwicklung wahr, wenn sie Lehrpersonen für die Qualifizierung zur PLP empfehlen und somit eine Eignungsentscheidung treffen.

### Berufspraktische Ausbildung von Lehrpersonen

Die berufspraktische Ausbildung ist ein zentrales Element der Lehrer:innenausbildung (z. B. Fraefel und Seel 2017). Darüber herrscht im deutschsprachigen Raum ein „Common Ground“ (Reusser und Fraefel 2017, S. 17). Hinsichtlich der Ausgestaltung bestehen nicht nur zwischen den Ländern vielmehr auch zwischen einzelnen Institutionen Unterschiede (ebd.). Nach Fraefel und Seel (2017, S. 7) lassen sich jedoch in der Schweiz, Deutschland und Österreich Trends erkennen. Diese neuen Gestaltungsformen umfassen, u. a. die Etablierung von Partnerschaftsstrukturen und Zusammenarbeitsformen zwischen Ausbildungsinstitution und Schulen, ko-konstruktive Ausbildungsformate und „die Schaffung hybrider Erfahrungs‑, Denk- und Handlungsräume, sogenannte ‚Third Spaces‘“ (Reusser und Fraefel. 2017, S. 18 ff.; auch Möller 2012). „Third spaces in teacher education are spaces where academic knowledge, practitioner knowledge, and the knowledge that exists in communities come together in new, less hierarchical ways, in the service of teacher learning“ (Zeichner 2010, S. 92). Die berufspraktische Ausbildung kann als „hybrider Erfahrungs- und Diskursraum“ (Reusser und Fraefel. 2017, S. 19) gesehen werden, der aus der Ausbildungsinstitution mit ihrem wissenschaftsbezogenen Wissen und der Schule mit dem Erfahrungswissen der SL und Lehrpersonen und dem Handlungsdruck zusammengesetzt ist (ebd., 2017, S. 19). Alle involvierten Akteur:innen sind notwendig, damit die berufspraktische Ausbildung gelingen kann. Hierzu braucht es partizipative Aushandlungs‑, Ko-Konstruktionsprozesse und eine von Symmetrie geprägte Kooperation zwischen den Akteur:innen (ebd. 2017, S. 19; Fraefel 2023). Nach Laros et al. (2024) sind jedoch die Beziehungen zwischen den Akteursgruppen von Asymmetrien geprägt. Die PLP scheinen die Erwartungen einerseits der Ausbildungsinstitution und andererseits der SL erfüllen zu müssen, ohne dass eine Kooperation bzw. Ko-Konstruktionsprozesse entstehen würden (ebd., S. 79). Eine Schlussfolgerung, die daraus gezogen werden kann, ist, dass die Rollen (und damit eingehenden Anforderungen) der Akteur:innen noch einmal überdacht und neu ausgerichtet werden müssten. Guillen und Zeichner (2018, S. 1) halten in diesem Zusammenhang fest: „More research is needed on how to both build and sustain these win-win partnerships between local schools and pre-service teacher preparation programs“.

### Aufgabenbereiche von Schulleitenden in der Ausbildung von Lehrpersonen

In der Regel sind in der Schweiz die Aufgaben zur berufspraktischen Ausbildung von Lehrpersonen in den Berufsaufträgen von Schulleitungen nicht definiert. Eine Ausnahme bilden die Kantone Graubünden^98^ und Luzern^97^ (vgl. Kanton Luzern, Dienststelle Volksschulbildung umsetzungen-dvs.lu.ch), welche im Berufsauftrag festschreiben, dass die SL genügend Praktikumsplätze und qualifizierte PLP zur Verfügung stellen müssen. Sowie werden sie im Kanton Luzern zur Zusammenarbeit mit der PH verpflichtet.

In verschiedenen Kantonen werden diverse Kooperations- und Partnerschulmodelle gelebt. In der Zusammenarbeit mit den PH werden dabei vertraglich unterschiedliche Komponenten geregelt, die jedoch je PH und kantonalen Zusammenarbeitsbündnissen variieren. Insgesamt berichtet Scherrer (2020, S. 333) von einem fehlenden Mandat der SL in der berufspraktischen Ausbildung von Lehrpersonen, sie würden sich jedoch trotzdem dafür einsetzen und interpretieren ihre Rolle in Abhängigkeit von ihrem Führungsverständnis. Die SL haben die Personalverantwortung und sind damit die Vorgesetzten der PLP (ebd., 2020; Laros et al. 2024). Sie wählen PLP aus und empfehlen sie den PHs (Scherrer 2020; Laros et al. 2024). Laros et al. (2024) berichten hier eher von Rollen der Reagierenden, welche den Wunsch der Lehrperson, PLP zu werden, berücksichtigen und bei auftretenden Konflikten während der Praktikumsphasen Ansprechperson sind. Nach Scherrer (2020) nehmen die SL eine planende Rolle ein, indem sie wissen, wann die Praktika stattfinden, und den Informationsfluss gewährleisten. Neben der Ansprechperson in schwierigen Praktikumssituationen sind sie nach Scherrer (2020) auch Ansprechperson, wenn Studierende besonders positiv auffallen, um sie für eine offene Stelle zu gewinnen. Scherrer (2020, S. 310 f.) nennt weitere Rollenfelder der SL in der berufspraktischen Ausbildung wie z. B. Entwicklungs‑/Weiterbildungsberater:in.

Dadurch übernehmen SL Funktionen in der Lehrer:innenausbildung. Ihre Wahrnehmung diesbezüglich ist allerdings sehr unterschiedlich, bspw. sehen gewisse SL die berufspraktische Ausbildung als Chance für Entwicklung der eigenen Schule, andere sehen sie als Dienstleistung für die PH (ebd., 2020, S. 322). Obwohl diese Ergebnisse interessant sind, beruhen sie auf wenigen Interviews, und es bleibt weitgehend empirisch ungeklärt, inwiefern SL eine (relevante) Funktion einnehmen oder sie sich eine Aufgabe hinsichtlich der Ausbildung des Berufsnachwuchses an den Schulen zuschreiben. Im Projekt PraLeB‑S wird dieser Frage in zwei Schritten nachgegangen: mit Experteninterviews (*N* = 19) und einer Fragebogenerhebung. Im vorliegenden Beitrag werden die Befunde aus den Experteninterviews vorgestellt.

## Forschungsfragen

Das Erkenntnisinteresse der hier dargelegten Teilstudie folgt drei Themenbereichen: 1) das Personalentwicklungsverständnis von SL, 2) die Anforderungen an PLP aus der Sicht von SL und 3) die Funktion bzw. Rolle der SL in der Lehrer:innenausbildung.

Konkret sollen folgende Fragen beantwortet werden:

1. *Welche Kriterien für die Auswahl von geeigneten PLP (Eignungskriterien) führen die SL an? Welche Qualifizierungsnotwendigkeiten und Möglichkeiten der Beförderung für die neue Rolle/Aufgabe als PLP sehen die SL? Wer trägt aus der Sicht der SL die Verantwortung für die Qualität der PLP-Tätigkeit?*
2. *Welche Anforderungen schreiben SL der PLP-rolle und -tätigkeit zu? Welche Unterstützungsleistung ergibt sich hieraus von Seiten der SL?*
3. *Welche Funktionen schreiben sich SL selbst im Kontext von Lehrpersonenausbildung zu? a) Bezogen auf die Studierenden und ihre Praktikumsaktivitäten, b) Bezogen auf die Kooperation mit der PH, c) Bezogen auf die Schule.*

## Design der Studie und Methodik

Im Rahmen des PraLeB‑S Projektes, das eine längsschnittliche Interviewstudie mit PLP und eine Interview- und Fragebogenstudie mit SL umfasst (Laros et al. 2024), werden mit 19 SL Expert:inneninterviews durchgeführt. Der Interviewleitfaden und die Analyse beziehen sich auf folgende Themenschwerpunkte (Auszug aus dem Interviewleitfaden):

1. Voraussetzungen für die Tätigkeit einer Lehrperson als PLP (Eignung, Kriterien)
  Wenn ich als Lehrperson an Ihrer Schule PLP werden möchte: Was wäre der Weg? Was müsste ich tun?
  Könnten Sie ganz konkret ein paar Kriterien nennen, die Sie bei Ihrer Entscheidung heranziehen bzw. herangezogen haben?
  Gibt es ein:e Kolleg:in bei der Sie die Entscheidung für die PLP-Funktion getroffen haben. Wie war dies konkret? Wie sind Sie bei der Auswahl vorgegangen?
  Was wären für Sie Gründe einer Lehrperson den Wunsch PLP zu werden zu verwehren?
2. Anforderungen von PLP aus Sicht der SL, Unterstützung und Qualifizierungsnotwendigkeiten
  PLP nehmen neben ihrer Lehrpersonenaufgabe eine zusätzliche Ausbilderinnen-Funktion wahr. Wo sehen Sie als SL Anforderungen?
  Was brauchen Ihrer Ansicht nach Lehrpersonen, um in diese (neue) Berufsrolle als Ausbildner:in hineinzuwachsen?
  Wie können Sie als SL Ihre PLP in ihrer Rolle unterstützen?
  Wie verschaffen sie sich ein Bild über die Qualität der PLP? Inwiefern ist der Einsatz von Lehrpersonen als PLP Teil der von Ihnen geführten Mitarbeitendengesprächen?
  Welche Möglichkeiten sehen Sie, damit die PLP die notwendigen Kompetenzen weiterentwickeln können?
  Wer ist aus ihrer Sicht zuständig für die Qualitätssicherung der PLP?
3. Funktion der SL in der Ausbildung
  Wie würden Sie Ihre Rolle als SL bezogen auf die Ausbildung von zukünftigen Lehrpersonen beschreiben? Wie sind Sie involviert?
  Manchmal gibt es ja auch Konflikte und Unstimmigkeiten zwischen den PLP und ihren Studierenden. Inwiefern werden Sie dann involviert? Wie verstehen Sie Ihre Aufgabe in solchen Situationen?
  Welchen Nutzen hat die Schule, wenn Studierende ihre Praktika dort absolvieren?
  Wo sehen Sie Möglichkeiten für einen Transfer von Ausbildungsinhalten der PH in Ihre Schule und in den Unterricht? Wie können Sie das unterstützen?
  Inwiefern gibt es Austausch zwischen ihnen und anderen SL, die Praktika bei sich anbieten? Wo ergeben sich für Sie Kontakte zur Pädagogischen Hochschule? Wie nehmen Sie die Zusammenarbeit mit Akteur:innen der Hochschule wahr?

Die Interviews haben eine durchschnittliche Dauer von ca. 60 min. Die Stichprobe (*N* = 19) setzt sich wie folgt zusammen: Vier Personen sind SL der Sekundarstufe und 15 weitere leiten eine Schule auf Primar- und Kindergartenstufe. Durchschnittlich sind sie 49 Jahre (zwischen 36 und 62 Jahre) alt und an ihrer Schule sind im Durchschnitt 42 Lehrpersonen (zwischen 8 und 90 LP), davon neun PLP (zwischen 3 und 30 PLP) beschäftigt. Sechs Schulen kooperieren als Praxiszentrum bzw. Kooperationsschule mit der PH.

Die Auswertung der transkribierten Interviews erfolgte nach einer inhaltlich strukturierenden qualitativen Inhaltsanalyse in Anlehnung an Kuckartz und Rädiker (2022, S. 104). Es wurde ein inhaltsanalytisch deduktiv und induktiv entwickeltes Kategoriensystem erarbeitet, bei dem die kodierten Sequenzen einer semantischen Einheit entsprechen und keine Doppel‑/Mehrfachnennungen sowie Gewichtungen vorgenommen wurden. In einem ersten Schritt wurden anhand des Interviewleitfadens und der Forschungsfragen deduktiv die Hauptkategorien der ersten und zweiten Ebene bestimmt (siehe Tab. 1). Zur Ermittlung der Kodierübereinstimmung wurde 20 % des Datenmaterials doppelt kodiert und die Cohens’ Kappa Werte ermittelt (Wirtz und Caspar 2002). In einem 2. Schritt erfolgte die Basiskodierung anhand der Hauptkategorien aller Interviews. In einem 3. Schritt wurden induktiv Unterkategorien auf der 3. und 4. Ebene erarbeitet. Hierfür wurden von den Kodierenden anhand von zwei Interviews Unterkategorien bestimmt und gemeinsam diskursiv festgelegt. Dieses Verfahren durchlief mehrere Schlaufen, bis keine zusätzlichen Kategorien mehr bestimmt werden konnten. In einem 4. Schritt wurden die restlichen Interviews mit allen Unterkategorien der 3. und 4. Ebene kodiert. In den Ergebnissen sind die Kategorien der deduktiven 1. und 2. Ebene und der induktiven 3. Ebene dargestellt. Insgesamt wurden 1481 Kodes gesetzt (auf einige Aspekte wird im Folgenden nicht eingegangen, wie bspw. der berufsbiographische Werdegang der SL).

**Tab. 1:** Deduktive Kodes der ersten und zweiten Ebene und Intercoderreliabilität

Teilbereich	Cohens’ Kappa	Ankerbeispiel
*1. Personalentwicklungsverständnis von SL*	0,88	–
1.1 Auswahlkriterien für zukünftige PLP	–	„ist dass jemand wo auch ich (.) finde dass sie ihre Arbeit qualitativ so gut macht dass (…) ich auch finde doch eine Studierende und ein Studierender hat ein Vorbild an dieser Person“ (SL_02)
1.2 Qualifikationsnotwendigkeiten und Möglichkeiten der Beförderung für die neue Rolle/Aufgabe als PLP (aus der Sicht von SL)	–	„(…) ich denke das ist es ist ja nicht (…) ein kompletter Rollenwechsel oder ein kompletter Berufswechsel //mhm// sondern es ist irgendwo (…) eine Erweiterung (.) (...) lerning by doing ist sicher eines der zentralen (…) Geschichten“ (SL_10)
1.3 Verantwortung für die Qualität der PLP-Tätigkeit aus der Sicht von SL	–	„und dass das Lehrer sind, denen man auch Praktikanten anvertrauen kann (.) (…)“ (SL_12)
*2. Anforderungen von PLP aus der Sicht der SL*	1,00	–
2.1 Anforderungen PLP-Funktion	–	„man muss schon (.) recht gut wissen, was im Moment eigentlich so gelehrt wird (…) wie kann man dann die (…) Studentinnen und Studenten da gut unterstützen“ (SL_05)
2.2 Unterstützung der PLP durch SL	–	„die zweite Unterstützung ist das man organisatorisch natürlich schaut, dass sie (…) möglichst entlastet sind von Zusatzaufgaben (…)“ (SL_15)
*3. Rolle und Funktionen SL in der Ausbildung*	0,78	–
3.1 Rolle der SL im Ausbildungskontext	–	„Ja ich habe (.) einfach den Anspruch, wenn die Studenten da sind, dass ich mit denen auch im Gespräch bin. (…)“ (SL_12)
3.2 Unterrichtsbesuche durchführen, wenn Studierende unterrichten	–	„Eben ich mache das Classroom walkthrough und dann ist es durchaus mal passiert, dass ich dann auch in (.) einen Unterricht einer Studierenden hineingeplatzt bin (…)“ (SL_18)
3.3 An Unterrichtsbesprechungen teilnehmen	–	„Ja ich glaube (.) dass (.) also da müsste ich wie eingeladen sein. (…) weil ich würde vielleicht einmal fragen (…) wärst du froh ich würde einmal hineinsitzen und dir auch ein Feedback geben (…)“ (SL_02)
3.4 Bei Konflikten einbezogen werden	–	„Ja: dann bin ich manchmal so bisschen Mediatorin (…) und in diesen Momenten sage ich mir immer wieder *ja* dort braucht es einfach auch beide Seiten anhören können einfach neutral sein in diesem Ganzen drin“ (SL_04)
3.5 Kontakte zur PH und Zusammenarbeit	–	„oder was es denn braucht (…) würde ich wirklich jemanden von der PH dann beiziehen und mich mit der Person absprechen (…)“ (SL_08)
3.6 Nutzen für Schule	–	„Also ich persönlich finde halt da bleibt man wie so im Puls der Zeit (…)“ (SL_05)
3.7 Vernetzung/Austausch zur schulpraktischen Ausbildung (schulübergreifend)	–	„Gut hier in (Ort) haben wir natürlich den Austausch unter uns Schulleitungen und wir wissen immer voneinander, wenn man irgendwo wieder jemand im Einsatz haben und tauschen uns auch aus (.)“ (SL_04)
3.8 Unterstützung von Transfer von Ausbildungsinhalten in die Schule	–	„Ich könnte sicher Plattformen Zeitgefässe bieten die beispielsweise einmal Mentorinnen Mentor ähm die Lehrpersonen informieren könnte (…)“ (SL_07)
*Gesamthaft über alle Codes*	*0,89*	–

*Anmerkungen.* Die induktiv generierten Unterkategorien werden in den Ergebnistabellen aufgeführt

Die Kodierungen wurden anhand des computergestützten Analysetools MAXQDA 24 (veröffentlicht 2023) durchgeführt. Die Darstellung der Ergebnisse folgen nach Kuckartz und Rädiker (2022, S. 116) einer quantitativen Logik.

## Ergebnisse

### Personalentwicklungsverständnis von SL

Das Personalentwicklungsverständnis setzt sich aus drei Aspekten zusammen (siehe Tab. 1, 1.): Zum einen werden die von den SL genannten Kriterien für die Auswahl von geeigneten PLP (Tab. 1, 1.1) kodiert und zum anderen die Qualifizierungsmöglichkeiten und -notwendigkeiten (Tab. 1, 1.2), damit Lehrpersonen die zusätzliche Rolle als Ausbilder:in bewältigen können. Zuletzt wird analysiert, wen die SL hierfür in der Verantwortung sehen (Tab. 1, 1.3).

Tab. 2 zeigt, dass die SL vielseitige Kriterien zur Auswahl von geeigneten PLP nennen.

**Tab. 2:** Kategorien, Häufigkeit ihrer Nennungen und Prozentwerte bez. Auswahlkriterien für zukünftige PLP (Tab. 1, 1.1)

	Ʃ	%
Unterrichtsqualität	74	32,9
Selbstkompetenzen	38	16,9
Motivation für Schule, Unterricht und für PLP-Tätigkeit	25	11,1
Möglichkeit zur beruflichen Weiterentwicklung	19	8,4
Entsprechende Kontextfaktoren	17	7,6
Sozialkompetenzen	17	7,6
Persönlichkeit	15	6,7
Begleitung von Studierenden	13	5,8
Subjektive Überzeugungen: aktuellen Strömungen angepasst	7	3,1
*Insgesamt*	*225*	*100*

Mit insgesamt 74 gesetzten Kodes werden Kriterien von Unterrichtsqualität am häufigsten von den SL für die Auswahl von zukünftigen PLP genannt. Darunter fallen Aspekte wie die Klassenführung oder die Individualisierung und Differenzierung, die jedoch unterschiedlich argumentiert werden, wie folgende Zitate zeigen:

„das andere [Kriterium] ist ähm wie gelingt ihm die Klassenführung wie geht er mit den Schüler (…) wenn ich das Gefühl habe, das funktioniert einigermassen (…) würde ich den empfehlen“ (SL_12)

Während die Fähigkeit zur Klassenführung eher als Ausgangspunkt für die Aufnahme von Studierenden aufgerufen wird, wird mit der Fähigkeit zur Individualisierung eine Vorbildfunktion für die Studierenden genannt:

„eigenverantwortliches Lernen umsetzen (…) und so das Individualisieren ich glaube dass das muss die Lehrperson ihnen wie auch zeigen können oder (…) das haben wir gar noch nicht alle selbst erfahren an ihrer Schulzeit (…) und das wäre sicher wichtig“ (SL_05)

Mit 38 Nennungen werden Selbstkompetenzen der Lehrperson am zweithäufigsten für die Auswahl für die PLP-Tätigkeit erwähnt. Hierzu gehören eine hohe Belastbarkeit und Reflexionsfähigkeit, die als wichtig erachtet werden. „*Also es wäre ja schön, wenn man so eine riesen Auswahl @hätte@ (…) wir schauen sehr stark momentan wirklich wie die Belastung ist bei der Lehrperson*“ (SL_04).

Ein weiteres Auswahlkriterium ist die Motivation für Schule, Unterricht und die Praxislehrpersonen-Tätigkeit (25 Nennungen): „*Freude Lust das zu machen ist eigentlich das (…) man muss es gerne machen wollen und auch eben gerne Schule geben (…)*“ (SL_13).

Zudem werden die Möglichkeit zur beruflichen Weiterentwicklung (19 Nennungen), entsprechende Kontextfaktoren wie z. B. eine problemlose Klassenzusammensetzung oder genügend Berufserfahrung (17 Nennungen), Sozialkompetenzen (17 Nennungen) wie die Fähigkeit zur Zusammenarbeit, Kommunikation und Kritikausübung, Persönlichkeit wie genügend „Reife“ und Gelassenheit (15 Nennungen). Die Fähigkeit zur Begleitung von Studierenden wie mit Erwachsenen Lernenden umgehen und Feedback geben fällt mit 13 Nennungen auf die zweitseltenst genannte Kategorie. Den Schluss bilden subjektive Überzeugungen der Lehrpersonen, sich an aktuellen Entwicklungen im Schulbereich zu orientieren (7 Nennungen).

#### Qualifizierungsnotwendigkeiten und Möglichkeiten der Beförderung für die neue Rolle/Aufgabe als PLP aus der Sicht von SL

Wie in Tab. 3 ersichtlich, sehen SL die Bereitschaft zum Austausch mit Kolleg:innen, die als PLP tätig sind, und mit den Studierenden als Voraussetzung für die Tätigkeit an (14 Nennungen).

**Tab. 3:** Kategorien, Häufigkeit ihrer Nennungen und Prozentwerte bez. Qualifizierungsnotwendigkeiten und Möglichkeiten der Beförderung für die neue Rolle/Aufgabe als PLP (aus der Sicht von SL) (Tab. 1, 1.2)

	Ʃ	%
Bereitschaft zum Austausch mit Kolleg:innen und Studierende	14	26,4
Weiterbildung für PLP-Tätigkeit an PH	10	18,9
Persönliche Voraussetzungen	8	15,1
Learning by Doing	7	13,2
Individuelle Weiterbildung	6	11,3
Weiterbildung intern an Schule	4	7,5
Gemeinsam mit PH Seminare leiten	2	3,8
Drei Säulen: PH-Ausbildung, PLP-Treffen, SL	1	1,9
Qualifikation kaum notwendig	1	1,9
*Insgesamt*	*53*	*100*

Eine Ausbildung für die PLP-Tätigkeit an der PH, um die Qualifikationen zur PLP zu erlangen und in die Rolle als PLP hineinzuwachsen, wird mit 10 Nennungen erwähnt. Notwendig sind hierfür gewisse persönliche Voraussetzungen wie Interesse an der Ausbildung von Lehrpersonen, Reflexionsfähigkeit und Offenheit (8 Nennungen). Dass die PLP-Tätgkeit in und durch Erfahrung gelernt wird („Learning by Doing“), findet sich mit 7 Nennungen, gefolgt von individuellen und intern an der Schule gehaltene Weiterbildungen. Wenig wurden ko-konstruktive Formen mit gemeinsam (PH und PLP) Seminare leiten und ein Zusammenspiel zwischen der Ausbildung an der PH, dem Austausch an PLP-Treffen und die Weiterbildung initiiert durch SL an Schule genannt. Dass Qualifikation kaum notwendig ist, wurde einmal ausgesagt.

#### Verantwortung für die Qualität der PLP-Tätigkeit aus der Sicht der SL

Auf die Frage, wen die SL in der Verantwortung sehen, wenn es um die Qualität der PLP-Tätigkeit geht, unterscheiden sich die Positionen der SL (siehe Tab. 4). 10 Nennungen sehen die Verantwortung bei der PH, 7 Nennungen betreffen eine geteilte Verantwortung zwischen der PH und der SL, 5 nennen die SL als Hauptverantwortliche und 2 Aussagen beziehen sich darauf, dass sie sich das noch nie überlegt haben.

**Tab. 4:** Kategorien, Häufigkeit ihrer Nennungen und Prozentwerte bez. der Verantwortung für die Qualität der PLP-Tätigkeit aus der Sicht von SL (Tab. 1, 1.3)

	Ʃ	%
PH	10	41,7
Geteilte Verantwortung zw. PH und SL	7	29,2
SL	5	20,8
Keine Ahnung	2	8,3
*Insgesamt*	*24*	*100*

### Anforderungen von Praxislehrpersonen

Ein zentrales Erkenntnisinteresse der Teilstudie war zu erfahren, welche Anforderungen SL in der Doppelrolle und in der Ausübung der Ausbilder:innentätigkeit sehen und wie sie hierfür Hand bieten können (siehe Tab. 1, 2.).

#### Welche Anforderungen schreiben Schulleitungen der Praxislehrpersonentätigkeit zu?

Am häufigsten werden die Begleitung und Förderung von Studierenden als erwachsene Lernende genannt (Tab. 5). Die Abgrenzung zwischen den Rollen als Klassenlehrperson und als Ausbildner:in wird als Anforderung am zweithäufigsten erwähnt. „*das ist manchmal so ein bisschen das Loslassen einfach so die grosse Verantwortung für die eigene Klasse (.) was ja eigentlich schön ist was aber eben auch (.) zu viel sein kann oder man muss auch loslassen können*“ (SL04) genannt. Die Beurteilung von Studierenden wird als Anforderung aufgrund der Antinomie, die sich auftut, angesprochen: „*Also ich finde dass man gleichzeitig wie Coach ist für die Studierenden (.) und aber auch (.) beurteilend bewertend noch ist. … Ja (.) das finde ich eigentlich die grösste Schwierigkeit*“ (SL03). Ausserdem werden die organisatorischen Anforderungen und das Selbstmanagement der PLP angesprochen. Das Rollenbewusstsein als PLP, die heute viel weniger hierarchisch gegenüber den Studierenden als früher auftritt, zu entwickeln, wird als weitere Anforderung wahrgenommen. Auch wird von problematischem Verhalten von Studierenden gegenüber Schüler:innen, Eltern und der PLP selbst als Anforderung gesprochen. Eine SL erzählt von Konkurrenzsituationen zwischen den Studierenden und der PLP, die sie als herausfordernd empfindet.

**Tab. 5:** Kategorien, Häufigkeit ihrer Nennungen und Prozentwerte bez. Anforderungen PLP-Funktion aus der Sicht von SL (Tab. 1, 2.1)

	Ʃ	%
Begleitung und Förderung von Studierenden	18	24,7
Abgrenzung Klassenlehrpersonen und Ausbildner:innenrolle	18	24,7
Beurteilung von Studierenden	15	20,5
Organisation und Selbstmanagement PLP	9	12,3
Rollenbewusstsein von PLP	7	9,6
Problematisches Verhalten gg. Schüler:innen, Eltern und PLP	5	6,8
Umgang mit Konkurrenzsituation	1	1,4
*Insgesamt*	*54*	*100*

#### Welche spezifischen Unterstützungsmassnahmen bieten die SL ihren PLP?

Angesichts der erkennbaren Bewusstheit der SL, dass die Lehrpersonen mit der Ausführung der PLP-Tätigkeit Anforderungen zu bewältigen haben, war es von Interesse, deren Unterstützungsangebote zu erfahren. Die meisten der Nennungen (siehe Tab. 6) weisen darauf hin, dass die SL erst bei erkennbarem Bedarf und wenn die PLP mit einem Anliegen aktiv auf die SL zugeht, Unterstützung bietet. An folgendem Beispiel deutet sich ein umfassendes Bewusstsein einer SL für ihre Unterstützung an:

„also ich habe festgestellt dass die (.) PLP *vor al*lem dann Unterstützung brauchen wenn es Probleme irgendwelcher Art gibt (.) das erste ist sicher dass ich (.) *da* bin um ihre Probleme anzuhören sie allenfalls zu beraten in schwierigen Situationen (2) *dann* (.) haben wir natürlich ab und zu Situationen mit Studierenden die (.) *nicht* (.) geeignet sind (.) und (.) das kommt das landet dann meistens bei *mir* (.) weil das gibt dann auch (.) also da kommen die PLP mit *Fra*gen wie sollen wir mit dieser jener Situation *um*gehen (.) habe *ich* dann ja auch wieder also da (.) fliesst bei mir dann auch wieder die Verantwortung für die (.) Schule als *Ga*nzes und die Schülerinnen und Schüler speziell (.) mit hinein (.) also (.) wurde auch schon die Situation ergeben als die PLP ans Themen*forum* musste und (.) ein Studierender allein unterrichten sollte dass ich einfach sagen musste, der (.) unterrichtet nicht allein im Turnen (.) das kann ich nicht verantworten.“ (SL_14).

**Tab. 6:** Kategorien, Häufigkeit ihrer Nennungen und Prozentwerte bez. Unterstützung der PLP durch SL (Tab. 1, 2.2)

	Ʃ	%
Unterstützung bei Bedarf	14	25,5
Unterstützung bei Problemen, Fragen	10	18,2
Unterstützung durch wertschätzende Haltung	8	14,5
Unterstützung kaum vorhanden	7	12,7
Regelmässiger Austausch	5	9,1
Organisatorische Unterstützung	4	7,3
Entlastung bieten	2	3,6
Unterstützung durch Weiterbildung im Team	2	3,6
Kleine Aufmerksamkeiten (z. B. Geschenke)	1	1,8
Informationen bereithalten	1	1,8
Einführung neuer PLP	1	1,8
*Insgesamt*	*55*	*100*

Mit 8 Nennungen wird eine wertschätzende Haltung als Ausdruck von Unterstützung genannt. 7 Nennungen beziehen sich darauf, dass bisher kaum Unterstützung durch die SL vorhanden sei.

### Rolle und Funktion der SL in der Ausbildung

Am häufigsten (Tab. 7) wird die Rolle als Vorgesetzte:r von PLP von den SL genannt: „*ich glaube auch wieder als Personalverantwortliche von den PLP (.) versuche ich zu unterstützen …*“ (SL09).

**Tab. 7:** Kategorien, Häufigkeit ihrer Nennungen und Prozentwerte bez. Rolle und Funktionen der SL im Kontext der Lehrpersonenausbildung (siehe Tab. 1, 3.)

	Ʃ	%
*Rollen der SL im Ausbildungskontext* (siehe Tab. 1, 3.1)	58	19,9
Vorgesetzte/r: Personalverantwortung für PLP, Organisation, Admin	20	–
Eröffner/in von umfassenden Erfahrungen für Studierende	15	–
Keine aktive/wichtige Rolle	9	–
Unterstützer/in bez. Support und Arbeitsklima der PLP	8	–
Im Hintergrund Agierende/r: informelle Gespräche mit Studierenden führen	3	–
*Unterrichtsbesuche durchführen, wenn Studierende unterrichten* (siehe Tab. 1, 3.2)	20	6,9
Unterrichtsbesuche als „walkthroughs“	4	–
Zufällige, nicht strukturierte Unterrichtsbesuche	3	–
Nur wenn gewünscht	3	–
Wunsch Unterrichtsbesuche durchzuführen, jedoch kaum Zeit	2	–
Keine Unterrichtsbesuche durchführen	2	–
Unterrichtsbesuche zur Vorbeugung von Konflikten	2	–
*An Unterrichtsbesprechungen teilnehmen* (siehe Tab. 1, 3.3)	11	3,8
Kein Einblick in Unterrichtsbesprechungen	9	–
Teilnahme an Unterrichtsbesprechungen	1	–
Bei schwierigen Situationen	1	–
*Bei Konflikten einbezogen werden* (siehe Tab. 1, 3.4)	41	14,1
SL einbezogen in Konfliktlösung	23	–
Verantwortlichkeit liegt nicht bei SL	13	–
SL nie in Konflikte involviert worden	5	–
*Kontakte zur PH und Zusammenarbeit* (siehe Tab. 1, 3.5)	48	16,5
Kontakt über PH-Mentor:innen (PH-Mitarbeitende, welche Studierende im Praktikum begleiten)	13	–
Kein oder kaum Kontakt/Zusammenarbeit	8	–
Kontakt über Fachpersonen/Dozierende der PH	7	–
Angenehme Zusammenarbeit	7	–
Kontakt bei organisatorischen Fragen über E‑Mail, Telefon	5	–
Zusammenarbeit im Konfliktfall	2	–
Während der SL-Ausbildung	2	–
Regelmässiger Austausch	1	–
Zusammenarbeit bei Fragen der Berufseignung	1	–
Zusammenarbeit zur Gewinnung von Schulen für berufspraktische Ausbildung	1	–
Austausch, wenn ein neues berufspraktisches Modell eingeführt wird	1	–
*Nutzen für Schule* (siehe Tab. 1, 3.6)	73	25,1
Studierende als Brücke zu aktuellen Themen	17	–
Profilierung gegenüber anderen Schulen	16	–
Unterrichtsentwicklung	9	–
Professionalisierung durch Anwesenheit von Externen angeregt	8	–
Austausch und Vernetzung zur eigenen Reflexion und Weiterentwicklung	6	–
Intensiveren Austausch mit PH als Partnerschule	6	–
Merkmale der Schulkultur	4	–
Abwechslung für Schüler:innen im Unterricht	4	–
Begegnung auf Augenhöhe: gemeinsam Lernen	2	–
Unterstüzung und Entlastung ausserhalb der Praktika durch Studierende	1	–
*Vernetzung/Austausch zur schulpraktischen Ausbildung (schulübergreifend)* (Tab. 1, 3.7)	20	6,9
Mit anderem Schulkreis der Gemeinde	11	–
Keine Vernetzung/kein Austausch	7	–
Mit anderen SL	2	–
*Unterstützung von Transfer von Ausbildungsinhalten in die Schule *(siehe Tab. 1, 3.8)	20	6,9
Durch Studierende ohne aktives Zutun von SL	6	–
Bereitstellung von Plattformen und Zeitgefässen	4	–
Transfer von PLP ins Team	4	–
Neuer Gedanke – noch nie überlegt	2	–
Transfer gestaltet sich schwierig	1	–
Durch längere Praktika	1	–
Teilnahme PLP oder SL an PH-Modulen	1	–
Durch Weiterbildungen	1	–

Mit 15 Nennungen sprechen die SL von der eigenen Rolle als Eröffner:in von umfassenden Erfahrungen für die Studierenden, die über das Unterrichten hinausgehen: „*Mir ist es schon wichtig, (…) dass sie auch eingebunden sind in eine Schule und dass es da auch noch zusätzliche Aufgaben gibt und mir ist es wichtig, dass sie die auch kennenlernen und auch so ein bisschen wissen ja Lehrer zu sein heisst nicht nur einfach ich und meine Klasse sondern auch wir und unsere Schule und da sehe ich meine Rolle darin dass sie auch einen Teil davon mitbekommen*“ (SL06). 9 Nennungen beziehen sich darauf, dass die SL keine aktive oder wichtige Rolle im Kontext der Ausbildung von Lehrpersonen haben.

Während des Praktikumszeitraums werden Unterrichtsbesuche, während die Studierenden unterrichten, nicht systematisch (insgesamt 20 Nennungen) durchgeführt, und die Mehrheit der Nennungen beziehen sich darauf, dass die SL keinen Einblick in die Unterrichtsbesprechungen zwischen Studierenden und PLP (11 Nennungen) haben. Am häufigsten werden die SL einbezogen, wenn Konflikte vorhanden sind (41 Nennungen).

Mit der PH kommen die SL am häufigsten über die Mentor:innen (PH-Mitarbeitende, welche die Studierenden im Praktikum begleiten) in Kontakt (14 Nennungen). 8 Nennungen berichten davon, dass kein Kontakt und keine Zusammenarbeit mit der PH stattfinden.

Einen Nutzen für die Schule sehen die SL, indem die Schule durch die Studierenden über aktuelle Themen informiert wird (17 Nennungen) und sie sich als Praktikumsschule gegenüber anderen Schulen profilieren können. Sie sehen weiterhin Vorteile in der Akquise von neuen, guten Lehrpersonen (16 Nennungen).

Im Zuge der Bildung von Partner- und Kooperationsschulen haben sich die betroffenen SL mit Schulen der gleichen Gemeinde vernetzt (11 Nennungen). „*Gut hier in (Ort) haben wir natürlich den Austausch unter uns SL und wir wissen immer voneinander wenn man irgendwo wieder jemand im Einsatz haben und tauschen uns auch aus (.) wie läuft es was sind die Erfahrungen wie reagiert auch das Team auf die Studierenden …*“ (SL04). 7 Nennungen beziehen sich darauf, dass sie sich bezüglich der schulpraktischen Ausbildung nicht vernetzen.

Bezüglich der Frage, ob SL den Transfer von Ausbildungsinhalten ins Schulteam aktiv unterstützen, beziehen sich 6 Nennungen darauf, dass hier kein aktives Zutun notwendig ist, da der Transfer automatisch über die Studierenden erfolge. Jeweils 4 Nennungen enthalten die Aussage, dass sie Plattformen und Zeit dafür zur Verfügung stellen oder dass PLP die Inhalte ins Team tragen. 2 Nennungen beziehen sich darauf, dass dies für sie ein neuer Gedanke sei.

## Diskussion

Die Personalentwicklung (Auswahl, Vorbereitung, Weiterbildung, Motivation) stellt einen wichtigen Aspekt von Schulqualität dar (Böckelmann und Mäder 2018; Terhart 2016) und bezieht auch die Tätigkeit der PLP mit ein. Die SL empfehlen den PHs zukünftige PLPs. Welche Kriterien sie zur Auswahl zukünftiger PLP anwenden, wurde bislang noch nicht untersucht. Die vorliegende Studie zeigt, dass sie eine breite Palette an Auswahlkriterien für zukünftige PLP aufführen, wobei das Kriterium der Unterrichtsqualität am häufigsten genannt wird. Zukünftige PLP müssen insbesondere gut Schule geben und hierin als Vorbild dienen können. Kritisch wird jedoch angemerkt, dass häufig die Möglichkeit zur Auswahl im Kollegium fehlt. Aus diesem Grund wird möglicherweise die Motivation bzw. der Wunsch zur Übernahme der PLP-Tätigkeit als vordergründiges Kriterium genannt, was die Frage der Eignung im Bedarfsfall überschattet.

Für die Qualifizierung als PLP werden häufiger personale Eigenschaften, die die PLP bereits mitbringt, wie die Bereitschaft zum Austausch mit Kolleg:innen, welche ebenso als PLP tätig sind, oder die Reflexionsbereitschaft genannt. Nicht von allen Befragten wird die Weiterbildung an der PH als wichtig erachtet.

Für die Qualitätssicherung sehen die SL mehrheitlich die PH in der Verantwortung. Die geteilte Verantwortung im Sinne des „Third Space“ nach Reusser und Fraefel (2017) findet keine Mehrheit in den Antworten der SL.

Nach Košinár (2023) können aus Anforderungen Entwicklungsaufgaben und Unterstützungsangebote abgeleitet werden. Welche Anforderungen stellen sich den PLP aus der Sicht der SL? Die SL sehen *Anforderungen von PLP* im Begleiten und Beurteilen der Studierenden und im Abgeben der Klassenführung. Unterstützung bieten sie dahingehend, dass sie, wenn PLP mit Fragen und Problemen auf sie zukommen, beratend zur Verfügung stehen und der PLP Wertschätzung entgegenbringen. Von aktiver, progressiver Unterstützung wird weniger berichtet.

Nach Reusser und Fraefel (2017) kann die berufspraktische Ausbildung als „hybrider Erfahrungs- und Diskursraum“ (S. 19) gesehen werden, indem alle beteiligten Akteur:innen für eine gelingende Ausbildung notwendig sind. Wie nehmen die SL ihre Funktion in der Ausbildung von Lehrer:innen wahr? Sie schreiben sich am häufigsten die Rollen als Verantwortliche:r für die Personalentwicklung der PLP und als Eröffner:in ausserunterrichtlicher Erfahrungsräume für Studierende zu. Von einer Zusammenarbeit zwischen allen an der berufspraktischen Ausbildung beteiligten Akteur:innen wird wenig oder nur vereinzelt berichtet. Die Kontakte zueinander scheinen eher zufällig bspw. über die Mentor:innen der PH, die vor Ort an der Schule sind, oder durch „walkthroughs“, wenn die Studierenden in den Klassen der PLP am Unterrichten sind, zustande zu kommen. Eine Ausnahme findet sich bei den SL, die Teil einer Partner- oder Kooperationsschule der PH sind. Die geringe Eingebundenheit von SL verwundert angesichts der Tatsache, dass SL von vielen Vorteilen und Entwicklungsmöglichkeiten, wenn sie als Schule in die berufspraktische Ausbildung integriert sind, berichten: Studierende bringen aktuelle Inhalte mit in den Unterricht und an die Schule, was zu ihrer Weiterentwicklung beiträgt. Wie bereits Scherrer (2020) zeigt, können sich Schulen mit der Teilnahme an der berufspraktischen Ausbildung gegenüber anderen Schulen profilieren und potenziell gute zukünftige Lehrpersonen finden.

Die vorliegenden Ergebnisse ergänzen die noch sehr dünne Datenlage zur spezifischen Rolle der SL in der berufspraktischen Ausbildung. Als wichtige Akteur:innen im komplexen Zusammenspiel von Schule vor Ort und Hochschule ist ihre Sichtweise mitentscheidend für die weitere Entwicklung und Professionalisierung der Ausbildung von Lehrer:innen. Die SL sind sich ihrer personalentwicklungsbezogenen Verantwortung bewusst. Bezogen auf die Personalentwicklung der PLP kommt ein weiterer Akteur ins Spiel: die berufspraktische Ausbildung der PH (bpA), die Anforderungen an die PLP-Tätigkeit stellt. Darüber nachzudenken, wie sich die Personalentwicklung der PLP konkret auch in der Zusammenarbeit mit der PH gestalten soll, ist notwendig, damit in diesem Bereich die Grauzonen (Laros et al. 2024) der Zusammenarbeit gelichtet werden können. Hierzu ist das gegenseitige Verstehen der Anliegen der involvierten Akteur:innen (PLP, bpA und SL), die jeweils unterschiedliche Verantwortungen in der berufspraktischen Ausbildung tragen, als erster Schritt wünschenswert. Aus den Interviews wird ersichtlich, dass die Anliegen der PH und der PLP im Bewusstsein der SL mehrheitlich (noch) gar nicht angekommen sind, obwohl sie die Wichtigkeit der berufspraktischen Ausbildung anerkennen. Dies liegt auch daran, dass die SL (mit Ausnahmen) kein Mandat hierfür haben (vgl. Scherrer 2020). Ein „offizielles“ Mandat könnte jedoch helfen, entsprechende Thematik ins Bewusstsein zu rücken, da auch in der vorliegenden Studie SL (9 Nennungen) berichten, dass sie keine aktive Rolle in der bpA hätten. Hierfür wäre innerhalb der Kantone ein intensiverer Austausch notwendig, der die Rollen und Zusammenarbeit mit allen Beteiligten diskutiert und bezüglich Umsetzbarkeit prüft.

Die Limitationen der vorliegenden Studie beziehen sich auf das inhaltsanalytische Verfahren mit einer kleinen Stichprobe. Die quantitativ ausgerichtete Inhaltsanalyse stellt eine Reduktion des Bedeutungsinhalts dar und ist durch die wenig ausgeprägten theoretischen Grundlagen als explorativ zu betrachten. Ausserdem ist die kleine Stichprobe mit 19 SL unterschiedlicher Stufen und Schulgrössen heterogen zusammengesetzt, was eine Generalisierbarkeit der Aussagen zusätzlich einschränkt.

## Footnotes

1 Wir beziehen uns auf die Ausbildung zur Kindergarten‑, Primar- und Sekundarlehrperson (Sek I).

99 Praxislehrpersonen sind in der Schweiz diejenigen Lehrpersonen, die angehende Lehrpersonen während ihrer obligatorischen Praktika im Rahmen ihres Studiums an den Schulen begleiten, unterstützen und bewerten (für Deutschland: Mentor: innen).

2 SNF-Projekt „Die Praxislehrperson als Lehrerbildner:in: Orientierungen und Handlungspraxis in der Erfüllung des doppelten Berufsauftrags Lehrperson und Ausbildner:in – eine berufsbiographische Längsschnittstudie unter Einbezug der Schulleitungsperspektive“, Projektnummer 207803.

3 LCH Leitbild: Berufsleitbild für Lehrerinnen und Lehrer https://www.lch.ch/lch/grundlagen/berufleitbild-und-berufsethik-lch [Stand: 12.11.2025].

98 vgl. VSLGR Verband Schulleiternnen und Schulleiter Graubünden https://www.vslgr.ch/schule-leiten/beruf.html#submenu [Stand:12.11.2025]; https://www.gr.ch/DE/institutionen/verwaltung/ekud/avs/Volksschule/FO_Praktikumsplaetze_Weisungen_de.pdf [Stand:12.11.2025]

97 vgl. Kanton Luzern, Dienststelle Volksschulbildung, https://umsetzungen-dvs.lu.ch/book/670e7259a1001fd6fc020af4/670e73a9a1001fd6fc020af7 [Stand: 12.11.2025]
